# Road tunnel-derived coarse, fine and ultrafine particulate matter: physical and chemical characterization and pro-inflammatory responses in human bronchial epithelial cells

**DOI:** 10.1186/s12989-022-00488-5

**Published:** 2022-07-04

**Authors:** Tonje Skuland, Vegard Sæter Grytting, Marit Låg, Rikke Bræmming Jørgensen, Brynhild Snilsberg, Daan L. A. C. Leseman, Alena Kubátová, Jessica Emond, Flemming R. Cassee, Jørn A. Holme, Johan Øvrevik, Magne Refsnes

**Affiliations:** 1 Division of Climate and Environmental Health, Department of Air Quality and Noise, Norwegian Institute of Public Health, PO Box 222, 0213 Skøyen, Oslo, Norway; 2 Department of Industrial Economics and Technology Management, Norwegian University of Science and Technology, NTNU, Trondheim, Norway; 3 Norwegian Public Roads Administration, Trondheim, Norway; 4 National Institute for Public Health and the Environment – RIVM, PO Box 1, 3720 BA Bilthoven, The Netherlands; 5 Institute for Risk Assessment Sciences, Utrecht University, Utrecht, The Netherlands; 6 Department of Chemistry, University of North Dakota, Grand Forks, ND USA; 7 Division of Climate and Environmental Health, Norwegian Institute of Public Health, PO Box 222, 0213 Skøyen, Oslo, Norway; 8 Department of Biosciences, Faculty of Mathematics and Natural Sciences, University of Oslo, PO Box 1066, 0316 Blindern, Oslo, Norway

**Keywords:** Urban air particulate matter, Mineral particles, Stone particles, Organic carbon, Oxidative potential, Epithelial lung cells, Cytokines

## Abstract

**Background:**

Traffic particulate matter (PM) comprises a mixture of particles from fuel combustion and wear of road pavement, tires and brakes. In countries with low winter temperatures the relative contribution of mineral-rich PM from road abrasion may be especially high due to use of studded tires during winter season. The aim of the present study was to sample and characterize size-fractioned PM from two road tunnels paved with different stone materials in the asphalt, and to compare the pro-inflammatory potential of these fractions in human bronchial epithelial cells (HBEC3-KT) in relation to physicochemical characteristics.

**Methods:**

The road tunnel PM was collected with a vacuum pump and a high-volume cascade impactor sampler. PM was sampled during winter, both during humid and dry road surface conditions, and before and after cleaning the tunnels. Samples were analysed for hydrodynamic size distribution, content of elemental carbon (EC), organic carbon (OC) and endotoxin, and the capacity for acellular generation of reactive oxygen species. Cytotoxicity and pro-inflammatory responses were assessed in HBEC3-KT cells after exposure to coarse (2.5–10 μm), fine (0.18–2.5 μm) and ultrafine PM (≤ 0.18 μm), as well as particles from the respective stone materials used in the pavement.

**Results:**

The pro-inflammatory potency of the PM samples varied between road tunnels and size fractions, but showed more marked responses than for the stone materials used in asphalt of the respective tunnels. In particular, fine samples showed significant increases as low as 25 µg/mL (2.6 µg/cm^2^) and were more potent than coarse samples, while ultrafine samples showed more variable responses between tunnels, sampling conditions and endpoints. The most marked responses were observed for fine PM sampled during humid road surface conditions. Linear correlation analysis showed that particle-induced cytokine responses were correlated to OC levels, while no correlations were observed for other PM characteristics.

**Conclusions:**

The pro-inflammatory potential of fine road tunnel PM sampled during winter season was high compared to coarse PM. The differences between the PM-induced cytokine responses were not related to stone materials in the asphalt. However, the ratio of OC to total PM mass was associated with the pro-inflammatory potential.

**Supplementary Information:**

The online version contains supplementary material available at 10.1186/s12989-022-00488-5.

## Introduction

Air pollution particulate matter (PM) is one of the largest environmental risk factors to human health and all-cause mortality worldwide [1]. Inhalation of PM has been associated with development and/or exacerbation of cardiovascular diseases and various airway diseases such as asthma, chronic obstructive pulmonary disease (COPD) and lung cancer, as well as increased susceptibility towards respiratory infections [2–5]. Different classes of PM may induce specific airway pathologies, illustrating that PM-induced airway diseases are influenced by PM characteristics such as particle sizes and chemical composition [6]. Pulmonary inflammation is regarded as a key event contributing to the adverse health effects of ambient PM, and there is strong support for a causal relationship between induction of pro-inflammatory responses in the airway mucosa and development or exacerbation of respiratory disease by PM exposure [4, 5, 7]. The chemokine CXC-motif ligand 8 (CXCL8), interleukin (IL)-6, IL-1α and IL-1β are important cytokines in lung inflammation. The main role of CXCL8 is to attract neutrophilic cells to the site of damage in vivo, and subsequently engulf PM, microbes and damaged cells, and is thus an important part of the defence system in the airways. IL-1α, IL-1β and TNF-α are pro-inflammatory cytokines involved in the orchestration of CXCL8 and a range of other cytokines during inflammation [8, 9]. Reactive oxygen species (ROS) have been regarded as important determinants in PM-induced inflammation, and the ability to generate ROS in cell-free systems has been suggested as a proxy of particle toxicity [10, 11], although consistent correlations with biological effects are still lacking [12, 13]. The inflammatory responses to ambient PM have been related to a variety of PM constituents like organic carbon (OC), including polycyclic aromatic hydrocarbons (PAHs), as well as metals. However, the contribution of different particle constituents is highly dependent on the PM source, such as traffic, domestic heating and industrial processes, the cell types and endpoints examined [14–18].

PM derived from traffic comprises of a mixture of particles from fuel combustion and from abrasion of road pavement, tires and brakes [19, 20]. Diesel exhaust particles (DEP) generated by combustion of diesel fuel are the most studied source of traffic-derived PM. These particles are often found as aggregates of various sizes and consist of a carbon core with adhered soluble organic carbon (OC) and metals constituents that to a varying extent are considered to mediate DEP-induced inflammatory responses [21]. Particles generated from road wear consist primarily of mineral particles from the stone aggregate material, as well as organic components from bitumen [22]. Strict regulatory policies in EU have led to introduction of new filter and engine technology, new fuels, and an increased prevalence of electric vehicles. Due to this development the emissions from diesel engines have been substantially reduced over the last decades. Accordingly, the relative contribution of wear PM in traffic-derived PM has increased steadily, and this development will continue in the future [23].

PM is usually divided in three size fractions based on its aerodynamic diameter. Coarse PM consists of particles with a diameter between 10 and 2.5 μm, while fine PM in the present study is defined as particles between 2.5 and 0.18 μm, and ultrafine PM as particles below 0.18 μm. Wear PM has mostly been linked to the coarse fraction, but may also be a constituent of the fine fraction [7, 24, 25]. In comparison, combustion particles have mostly been associated with the fine and ultrafine fractions [26]. Although their effects may overlap, coarse PM has mainly been associated with respiratory effects, whereas the fine and ultrafine PM are more strongly linked to cardiovascular effects [7]. The potency of the different size fractions to cause adverse respiratory effects has been explored in epidemiological studies [27–29], in clinical human studies and in animal studies [14, 30–32], in addition to cell culture studies [11, 15, 17, 18, 30, 31, 33–38]. Several experimental studies have found that coarse ambient PM is more potent and induces higher pro-inflammatory responses than fine and ultrafine PM when compared at equal mass. This has mostly been attributed to high content of endotoxins [11, 15, 18, 30, 33, 35]. Endotoxins like lipopolysaccharide (LPS) are known to stimulate pro-inflammatory responses [39], and may enhance responses to PM at low concentrations [40].

The levels of ambient PM in Nordic countries are generally low. However, the levels of coarse PM may be high during winter season due to the use of studded tires, which generates more PM from road abrasion and resuspension of road dust than friction tires [41]. Source apportionment studies show that wear PM constitutes a high proportion of traffic-related PM [42, 43] and represent a major environmental challenge. Asphalt consists of 95% stone aggregates, with the remaining 5% consisting of bitumen and other additives [25]. Interestingly, PM from asphalt with different stone materials generated in a road wear simulator has been reported to induce pro-inflammatory responses with different potency in cell cultures of macrophages [24], suggesting a potential role for mineral composition in PM toxicity. In support of this, extensive studies by our research group have shown that stone- and mineral particles of different composition may vary in pro-inflammatory potential [44–47]. In early studies, particle samples with high content of feldspar minerals were the least potent, suggesting that it might be beneficial to use feldspar-rich stone types in the asphalt [46]. However, the full impact of using different stone materials for ambient PM toxicity is still largely unknown.

In the present study, traffic-related PM from roads paved with stone materials with different feldspar content was collected in two tunnels to reduce contamination with PM from other sources. PM was sampled both under humid and dry road surface conditions, and before and after road surface cleaning of the tunnels. The coarse, fine and ultrafine road tunnel PM samples were characterized for hydrodynamic size distribution, content of organic (OC) and elemental carbon (EC), endotoxin content and potential to induce acellular ROS. Furthermore, the pro-inflammatory potential of the tunnel PM samples was examined in human bronchial epithelial cells (HBEC3-KT). The responses of the tunnel PM samples were compared to the potency of particles generated from the respective stone materials used in the tunnels. Finally, the possible correlations between the different road tunnel PM characteristics and pro-inflammatory potential were analyzed.

## Results

Traffic-derived PM (coarse, fine and ultrafine fractions) were sampled inside two road tunnels during the winter season, both during humid and dry road surface conditions, and before and after road surface cleaning. The two tunnels Marienborg and Hell which are located in the Trondheim area, Norway (Additional file 1: Fig. S1) were paved with asphalt containing rhomb porphyry and quartz diorite, respectively.

### Characterization of the PM fractions

The mass of PM sampled per hour varied between the two tunnels and was lower during humid compared to dry conditions. In both tunnels, the mass of PM sampled was larger for the coarse fraction than for the fine and ultrafine PM during all conditions (Table 1). The levels of PM were consistently highest in the Hell tunnel for all size fractions, especially during dry conditions where the amounts of coarse, fine and ultrafine PM samples were 30-fold, 10-fold and 17-fold higher than in the Marienborg tunnel. The differences between the two tunnels were substantially lower during humid road surface conditions. Particles were also collected after road surface cleaning in both tunnels, a measure to reduce road dust. However, no substantial alterations in mass of sampled PM were observed after the road surface cleaning (Table 1).

**Table 1:** The hydrodynamic size distributions of road tunnel PM samples

Sample name (PM)	Size fraction	Sampled PM (mg/m^3^/h)	Median hydrodynamic size by mass (μm)	Median hydrodynamic size by number (μm)
Marienborg, dry conditions	Coarse (2.5–10 μm)	0.07	1.98	0.07
	Fine (0.18–2.5 μm)	0.05	1.50	0.07
	Ultrafine (< 0.180 μm)	0.03	1.72	0.07
Marienborg, humid conditions	Coarse (2.5–10 μm)	0.11	1.67	0.08
	Fine (0.18–2.5 μm)	0.05	1.52	0.08
	Ultrafine (< 0.180 μm)	0.05	1.15	0.07
Hell, dry conditions	Coarse (2.5–10 μm)	2.42	3.01	0.05
	Fine (0.18–2.5 μm)	0.49	1.56	0.06
	Ultrafine (< 0.180 μm)	0.50	2.22	0.07
Hell, humid conditions	Coarse (2.5–10 μm)	0.16	2.48	0.11
	Fine (0.18–2.5 μm)	0.06	2.07	0.08
	Ultrafine (< 0.180 μm)	0.04	0.79	0.07
Marienborg, after cleaning humid conditions	Coarse (2.5–10 μm)	0.18	2.22	0.06
	Fine (0.18–2.5 μm)	0.06	1.33	0.08
	Ultrafine (< 0.180 μm)	0.02	1.04	0.07
Hell, after cleaning dry conditions	Coarse (2.5–10 μm)	3.01	3.11	1.07
	Fine (0.18–2.5 μm)	0.66	1.49	0.08
	Ultrafine (< 0.180 μm)	0.46	1.44	0.07

Coarse, fine and ultrafine PM were sampled in two road tunnels (Marienborg and Hell) by a High-volume cascade impactor during dry and humid road surface conditions, respectively, and also after road surface cleaning. The amounts of the coarse, fine and ultrafine PM were weighed, and presented as mg PM/m^3^/h. The table also includes hydrodynamic size distributions as determined by the Disc centrifugation method and presented as median size related to PM mass and also to particle number

#### Hydrodynamic size distributions

**Fig. 1:**
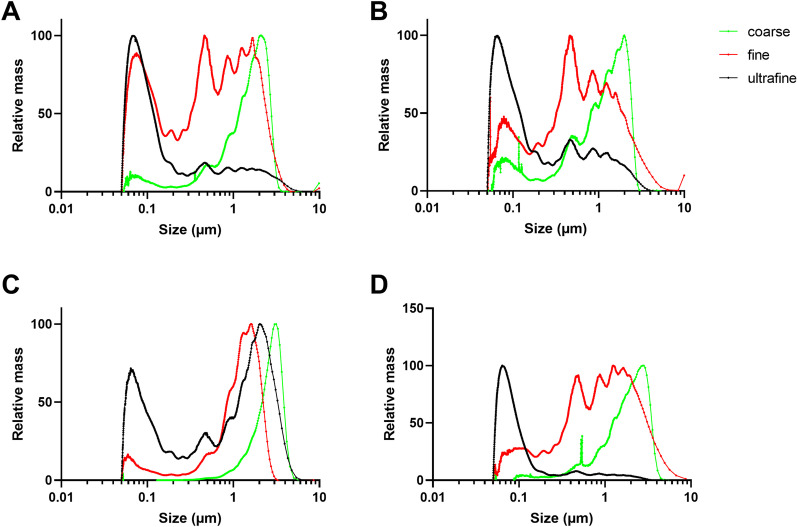
Hydrodynamic size distribution based on the relative mass of coarse, fine and ultrafine PM sampled in the Marienborg and Hell tunnels. **A** Marienborg PM, dry road surface conditions; **B** Marienborg PM, humid road surface conditions; **C** Hell PM, dry road surface conditions; **D** Hell PM, humid road surface conditions. The hydrodynamic size distributions were determined by the Disc centrifugation method, as described in “Materials and methods”, from representative analyses

Hydrodynamic size distributions of the coarse, fine and ultrafine PM samples after extraction from the filter and suspension in water are presented as the median size related to mass and number. On a mass basis, the data showed that the median sizes were largest for coarse PM samples, and smallest for the fine and ultrafine PM samples (Table 1). However, the median particle sizes were rather similar for the different PM fractions, possibly due to aggregation and agglomeration of particles during collection and processing. Although the differences were small, the size distribution profiles showed that the ultrafine fraction contained a substantial higher proportion of nano-sized (around 100 nm) particles compared to the coarse fractions, as well as most of the fine fractions (Fig. 1). Humid road surface conditions also seemed to affect size distributions on a relative mass basis, with a shift towards larger sizes for the fine and ultrafine PM sampled in the Marienborg tunnel (Fig. 1B), but not for the PM sampled in the Hell tunnel (Fig. 1D). Furthermore, road surface cleaning did not consistently affect the hydrodynamic size distribution profiles of the PM samples on a relative mass basis (data not shown). On a particle number basis, the median hydrodynamic sizes were substantially lower than on a mass basis, and relatively similar for the coarse, fine and ultrafine PM fractions (Table1). Nano-sized particles (< 100 nm) completely dominated particle counts for all PM samples, but with differences depending on the tunnel and road surface conditions (Additional file 2: Fig. S2). Road surface cleaning reduced the nano-sized peak substantially for the ultrafine PM fraction both from the Marienborg and Hell tunnels, whereas no consistent changes were observed for the fine and coarse PM fractions (data not shown).

#### Endotoxins

The levels of endotoxin (lipopolysaccharide; LPS) were low (< 1 EU/mg PM) in all tunnel PM samples (Table 2). Furthermore, exposure to LPS in the concentration range of 0.01–10 ng/mL (0.1–100 EU/mL) for 20 h induced no detectable cytokine responses in the HBEC3-KT cells (data not shown), suggesting that the impact of LPS on these cells is negligible.

**Table 2:** Endotoxin levels of road tunnel PM

Sample name (PM)	Size fraction	Endotoxins (EU/mg PM) mean ± SD
Marienborg, dry conditions	Coarse	0.29 ± 0.16
	Fine	0.24 ± 0.15
	Ultrafine	0.20 ± 0.08
Marienborg, humid conditions	Coarse	0.69 ± 0.36
	Fine	0.17 ± 0.08
	Ultrafine	0.19 ± 0.08
Hell, dry conditions	Coarse	0.61 ± 0.29
	Fine	0.25 ± 0.13
	Ultrafine	0.09 ± 0.09
Hell, humid conditions	Coarse	0.39 ± 0.26
	Fine	0.32 ± 0.18
	Ultrafine	0.43 ± 0.12
Marienborg, after cleaning humid conditions	Coarse	0.64 ± 0.37
	Fine	0.67 ± 0.35
	Ultrafine	0.84 ± 0.33
Hell, after cleaning dry conditions	Coarse	0.59 ± 0.37
	Fine	0.70 ± 0.44
	Ultrafine	0.42 ± 0.25

The table includes the coarse, fine and ultrafine PM from two tunnels sampled during both humid and dry road surface conditions, and after road surface cleaning. Endotoxin levels were determined by the LAL-method, as described in “Materials and methods”. The data are presented as the mean ± SEM of three analyses of the endotoxin levels

#### Acellular oxidative potential of the PM fractions

The capacity of particles to generate ROS in cell-free systems is regarded as a strong predictor of the potential health hazard of PM and has been proposed as a more relevant metric than PM mass. The oxidative potential (OP) of the tunnel PM samples was measured by electron spin resonance (OP^ESR^) and dithiothreitol (OP^DTT^) methods [10]. Correlation analysis showed that acellular ROS induced by the PM samples as measured by ESR and DDT was correlated (r = 0.947, *p* = 0.001, see also Additional file 3: Fig. S3). The coarse and fine PM samples from Marienborg road tunnel were almost equally redox active and exhibited 7–tenfold higher OP than the ultrafine PM samples. Humid and dry road surface conditions or road surface cleaning did not affect the acellular OP of the particles significantly. The PM sampled in the Hell tunnel, especially the coarse fraction, had a lower OP than the respective PM from the Marienborg tunnel. Also in the Hell tunnel, the ultrafine fraction had lowest OP in nearly all samples (Table 3).

**Table 3:** Oxidative potential (OP) of road tunnel PM

Sample name (PM)	Size fraction	OP^DTT^ (nmol DTT/ng*min)	OP^ESR^ (arbitrary units)
Marienborg dry conditions	Coarse	0.063	63,823
	Fine	0.068	78,288
	Ultrafine	0.007	24,389
Marienborg humid conditions	Coarse	0.070	73,555
	Fine	0.060	76,634
	Ultrafine	0.011	24,409
Hell dry conditions	Coarse	0.038	30,161
	Fine	0.043	37,216
	Ultrafine	0.013	13,035
Hell humid conditions	Coarse	0.026	32,764
	Fine	0.054	56,771
	Ultrafine	0.005	9330
Marienborg after cleaning humid conditions	Coarse	0.071	68,176
	Fine	0.062	69,284
	Ultrafine	0.010	26,630
Hell after cleaning dry conditions	Coarse	0.015	16,979
	Fine	0.043	39,594
	Ultrafine	0.026	24,184

Acellular ROS formation was determined by the dithiothreitol (DTT)-method and electron spin resonance (ESR)-method for the coarse, fine and ultrafine PM sampled from two tunnels during both humid and dry road surface conditions, and after road surface cleaning. For the DTT method the positive control was 0.1 and for the ESR the negative control was 2954, and the positive control was 54,119. The results are presented as the mean of three (OP^ERS^) and two (OP^DTT^) measurements

#### Organic and elemental carbon levels of the PM fractions

**Fig. 2:**
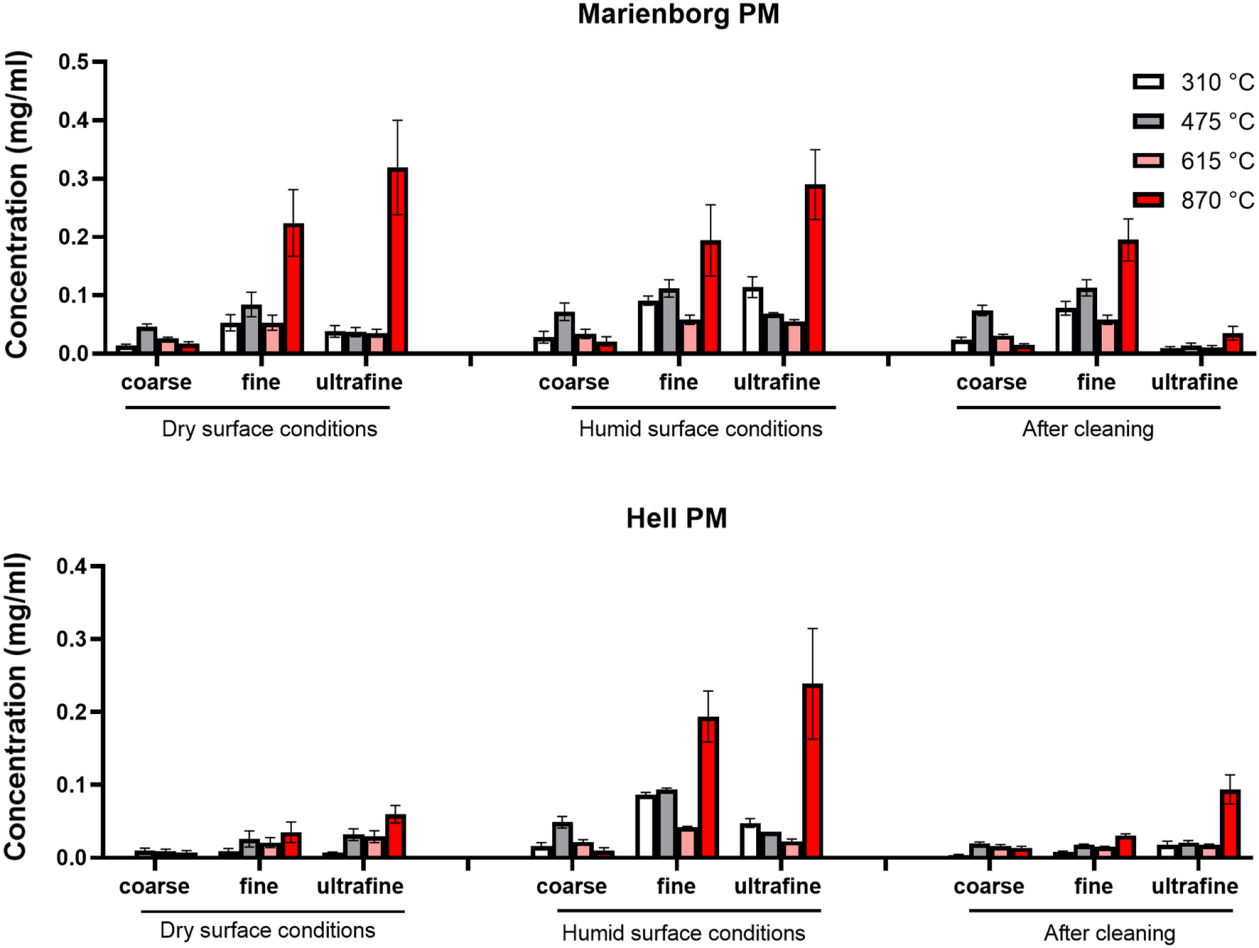
Organic carbon (OC) levels in PM sampled from the road tunnels. The figure includes the OC-content of coarse, fine and ultrafine PM samples from the two tunnels as obtained by increasing temperatures: 310 °C; 475 °C; 615 °C; 870 °C as indicated in the figure. The data represent the mean ± SEM of 3 analyses

The content of OC and EC was determined in all tunnel PM samples according to NIOSH protocol (Table 4). Most of the total carbon was OC, with no or very slight contribution of EC (< 1%). The only exception was the ultrafine PM from Marienborg tunnel sampled during humid conditions, which contained 120 µg EC/mg PM. The data showed that total OC varied between the size fractions, tunnels and road surface conditions. Overall, the fine and ultrafine PM samples seemed to contain higher OC levels than coarse PM samples, and all size fractions contained more OC when sampled during humid than dry conditions. The OC levels in particles sampled in the Marienborg tunnel were generally higher than the corresponding PM sampled in the Hell tunnel (Table 4). The OC content of the coarse and fine fraction were not profoundly affected by road surface cleaning in either tunnel. In contrast, cleaning was associated with a substantial reduction of OC in the ultrafine PM sampled in the Marienborg tunnel, but not in the Hell tunnel (Table 4). To further characterize OC from the PM samples, we used a method adopted from NIOSH temperature protocol allowing to distinguish different temperature fractions up to 870 °C (i.e., volatility and molecular weight; Fig. 2). The relative pattern of OC detected at various temperatures varied between coarse, fine and ultrafine fractions. Highest amounts of OC from coarse PM were detected at 475 °C. In contrast, both fine and ultrafine PM released highest amounts of OC at 870 °C.

**Table 4:** Elemental carbon (EC) and organic carbon (OC) levels, of road tunnel PM

Sample name (PM)	Size fraction	OC (Hg/mg PM)	EC (Hg/mg PM)
Marienborg, dry conditions	Coarse	104 ± 13	0.3 ± 0.6
	Fine	441 ± 110	4.3 ± 9.6
	Ultrafine	431 ± 107	1.8 ± 1.3
Marienborg, humid conditions	Coarse	157 ± 42	0.7 ± 0.8
	Fine	575 ± 60	0.0 ± 0.0
	Ultrafine	527 ± 38	119.8 ± 112.2
Hell, dry conditions	Coarse	28 ± 10	0.0 ± 0.0
	Fine	94 ± 37	0.0 ± 0.0
	Ultrafine	131 ± 29	0.0 ± 0.0
Hell, humid conditions	Coarse	98 ± 19	0.3 ± 0.5
	Fine	485 ± 14	0.0 ± 0.0
	Ultrafine	434 ± 32	3.2 ± 5.5
Marienborg, after cleaning humid conditions	Coarse	144 ± 17	0.7 ± 0.6
	Fine	483 ± 73	0.0 ± 0.0
	Ultrafine	69 ± 23	0.3 ± 0.5
Hell, after cleaning dry conditions	Coarse	55 ± 7	0.0 ± 0.0
	Fine	76 ± 6	0.4 ± 0.6
	Ultrafine	151 ± 29	2.6 ± 3.5

The table includes the coarse, fine and ultrafine PM sampled from two road tunnels during both humid and dry road surface conditions, and after road surface cleaning. EC and OC levels were determined by thermal optical analysis as described in “Materials and methods”, and presented as µg/mg PM. The data represent the mean ± SEM of 3 analyses

### Cytotoxic and pro-inflammatory responses in HBEC3-KT cells

In the present study, important cytokines such as chemokine CXC-motif ligand 8 (CXCL8), interleukin (IL)-6, IL-1α and IL-1β were examined to assess the pro-inflammatory potential.

#### Concentration–response relationship of pro-inflammatory effects of PM size fractions

**Fig. 3:**
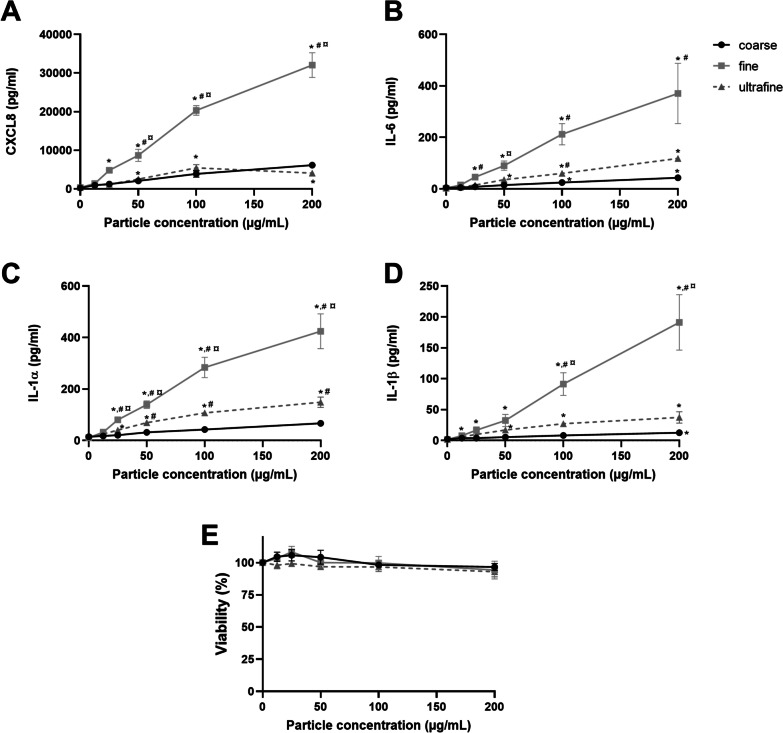
Concentration-dependent cytokine responses in HBEC3-KT cells after exposure to PM sampled from a road tunnel. The cells were exposed to coarse, fine and ultrafine PM sampled from the Marienborg road tunnel during dry road surface conditions. The cells were exposed to 0–200 µg PM/mL (0–20.8 µg/cm^2^) for 20 h. The cytokine release was analysed by ELISA. **A** CXCL8; **B** IL-6; **C** IL-1α; **D** IL-1β. The cell viability was determined by AlamarBlue (**E**). The data represent the mean ± SEM of 3 experiments. Statistical analysis was performed by two-way ANOVA (repeated measured) with Dunnett’s or Tukey multiple comparison test. *Significantly different compared to control, # significantly different from coarse PM, ¤ significantly different from ultrafine PM, *p* < 0.05

Initially, the pro-inflammatory and cytotoxic potentials of the coarse, fine and ultrafine PM fractions sampled in the Marienborg tunnel upon dry road surface conditions were examined by exposing HBEC3-KT cells to 0, 12.5, 25, 50, 100 and 200 µg/mL (equivalent to 0, 1.3, 2.6, 5.2, 10.4 and 20.8 μg/cm^2^) of the respective PM fractions for 20 h. The release of the pro-inflammatory cytokines CXCL8, IL-6, IL-1α and IL-1β was markedly increased, with significant and progressive increases for the fine road tunnel PM fraction from 25 µg/mL, and substantially higher than for the coarse and ultrafine PM fraction. The response to the coarse PM fraction was similar to ultrafine PM fraction for CXCL8, but significantly lower for IL-6, IL-1α and IL-1β (Fig. 3). None of the particle fractions significantly reduced the viability of the cells for this concentration-range, as measured by the AlamarBlue assay (Fig. 3E). Similarly, no significant increase in lactate dehydrogenase (LDH) release was detected (data not shown). The concentration–response relationships of PM from the Hell tunnel, as sampled upon dry conditions, induced similar patterns as for PM from the Marienborg tunnel, although with responses of less magnitude (data not shown).

#### Comparison of pro-inflammatory potential of all the sampled PM

**Fig. 4:**
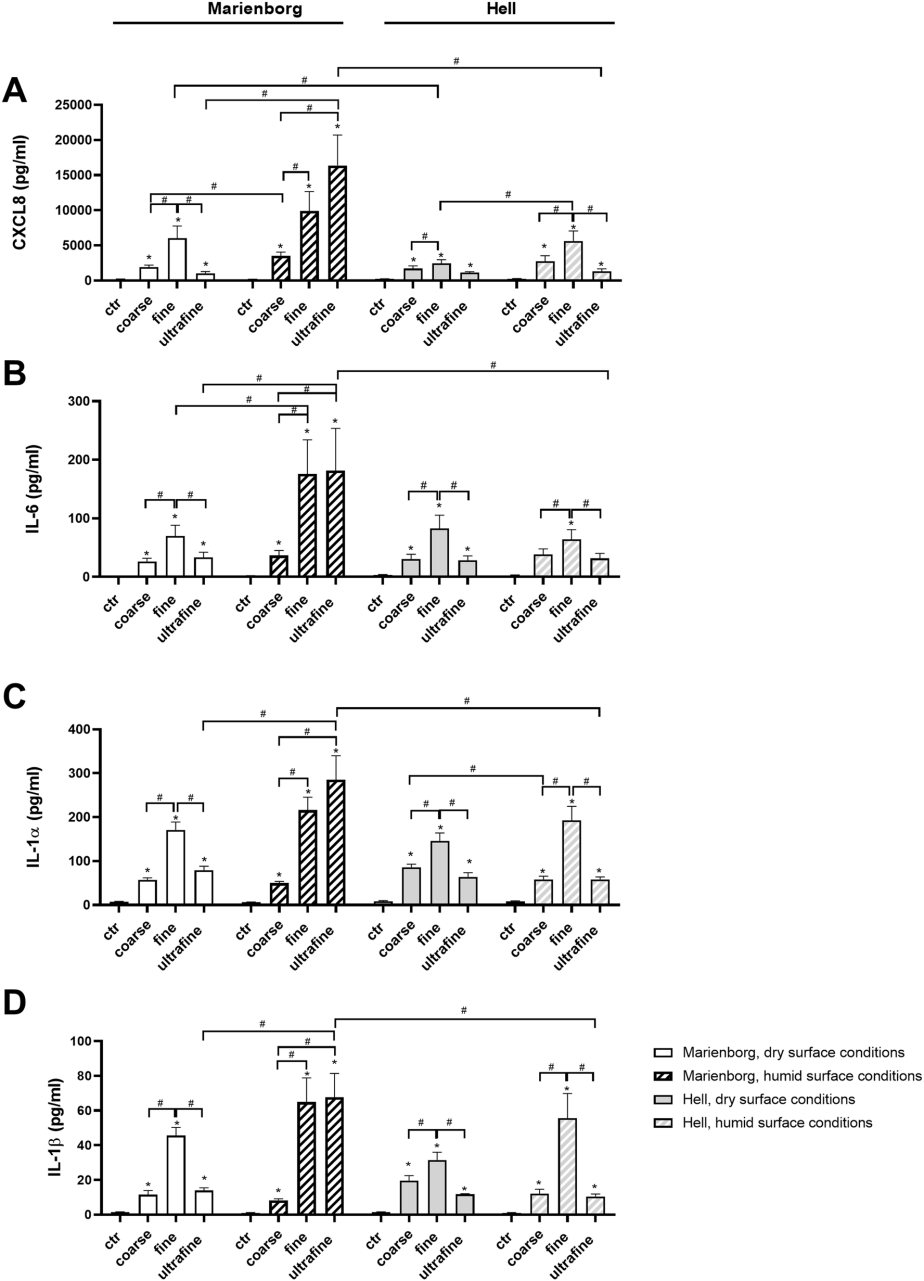
Cytokine responses in HBEC3-KT cells after exposure to PM samples from two road tunnels. The cells were exposed to coarse, fine and ultrafine PM samples from the Marienborg and Hell road tunnels during dry and humid road surface conditions. The cells were exposed to 100 µg/mL (10.4 µg/cm^2^) PM samples for 20 h. The cytokine release was analysed by ELISA. **A** CXCL8; **B** IL-6; **C** IL-1α; **D** IL-1β. The data represent the mean ± SEM of 5 experiments. Statistical analysis was performed by two-way ANOVA (repeated measurements) with Tukey’s multiple comparisons test. *Significantly different from control, # significantly different between groups, *p* < 0.05

Next, we exposed the HBEC3-KT cells to 100 µg/mL (10.4 ug/cm^2^) of the coarse, fine, and ultrafine PM fractions from the 6 different samples, including both road tunnels, both during dry and humid road surface conditions, and after road surface cleaning. None of the PM samples induced cytotoxicity at this concentration (data not shown). A similar pattern of potency between the different PM samples was observed for most cytokine responses. In agreement with Fig. 3, the fine PM fraction appeared to be the most potent for all samples, with significantly higher responses than coarse and ultrafine PM fractions. However, the only exception was the ultrafine PM sampled in the Marienborg tunnel during humid conditions, which were similar in potency to the fine PM fraction and significantly higher than the particles sampled during dry conditions (Fig. 4A-D). Conversely, responses induced by the ultrafine PM fraction from the Hell tunnel were low and seemed unaffected by the road surface conditions (Fig. 4A-D). The cytokine responses to fine PM fractions tended to be highest during humid conditions, but the differences were only statistically significant for CXCL8 in the Hell tunnel and IL-6 in the Marienborg tunnel. Overall, the response patterns were rather similar for PM samples from the two tunnels, but significantly higher responses were observed for ultrafine PM sampled in Marienborg tunnel during humid conditions compared to corresponding particles sampled in the Hell tunnel (Fig. 4A-D). In addition, fine PM sampled during dry conditions in the Marienborg tunnel induced a significantly higher CXCL8 response than fine PM from the Hell tunnel (Fig. 4A). The PM sampled after road surface cleaning in both tunnels were also examined (Additional file 4: Fig. S4). Reductions in pro-inflammatory potential were only observed for the ultrafine PM sampled in the Marienborg tunnel, reaching statistical significance for IL-1α and IL-1β.

#### Comparison of pro-inflammatory responses to road tunnel PM and stone particles

**Fig. 5:**
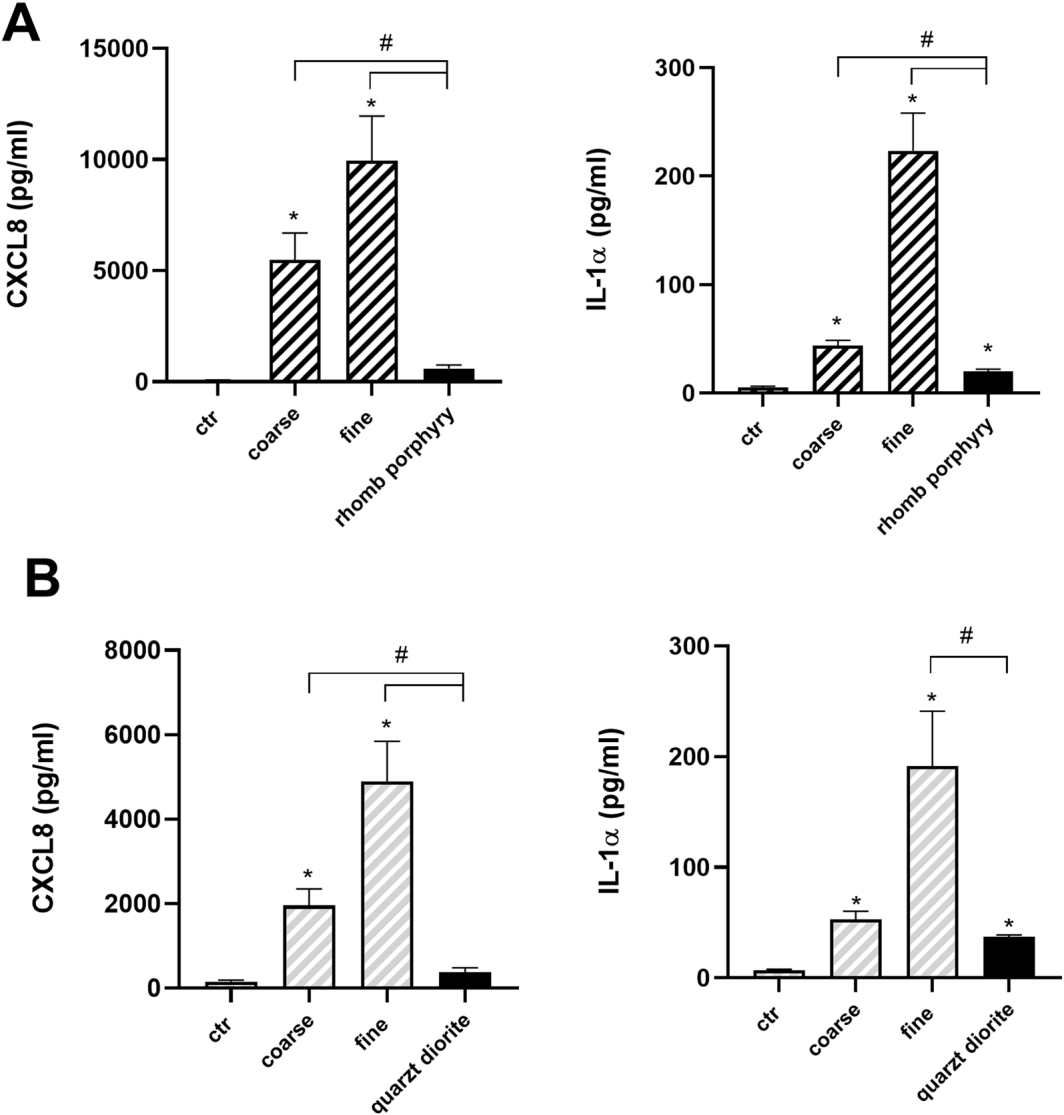
Cytokine responses in HBEC3-KT cells after exposure to PM samples from two road tunnels in comparison to stone particles from the respective tunnels. The cells were exposed to coarse, fine and ultrafine PM samples from the Marienborg (**A**) and Hell (**B**) road tunnels, respectively, during humid road surface conditions. The cells were also exposed to rhomb porphyry and quartz diorite for 20 h, at same concentration as the tunnel PM of 100 µg/mL (10.4 µg/cm^2^). The CXCL8 and IL-1α release were analysed by ELISA. The data represent the mean ± SEM of 5–7 experiments. *Significantly different from control, # significantly different from coarse, fine and ultrafine PM. One-way ANOVA with Tukey’s multiple comparison test, repeated measurements with p < 0.05

The pro-inflammatory responses to traffic-derived coarse and fine PM from the Marienborg and Hell tunnels were compared to the equivalent concentrations (100 µg/mL; 10.4 µg/cm^2^) of rhomb porphyry and quartz diorite, the respective stone materials used in pavement of the tunnels. The stone materials induced lower CXCL8 and IL-1α responses in the HBEC3-KT cells compared to the road tunnel PM, although the differences in magnitude depended on the PM size fractions and tunnel. For the Marienborg tunnel, the stone material rhomb porphyry induced significantly lower cytokine responses than coarse and fine PM size fractions sampled during humid conditions (Fig. 5A). For CXCL8, the coarse and fine PM samples induced responses that were approximately 10-fold and 15-fold higher than the rhomb porphyry (Fig. 5A). For the Hell tunnel, the stone material quartz diorite induced a CXCL8 response that was significantly lower than for the coarse and fine PM samples (Fig. 5B). For IL-1α a significantly lower response was only observed compared to the fine PM.

#### Correlations between particle characteristics and bioactivity

**Fig. 6:**
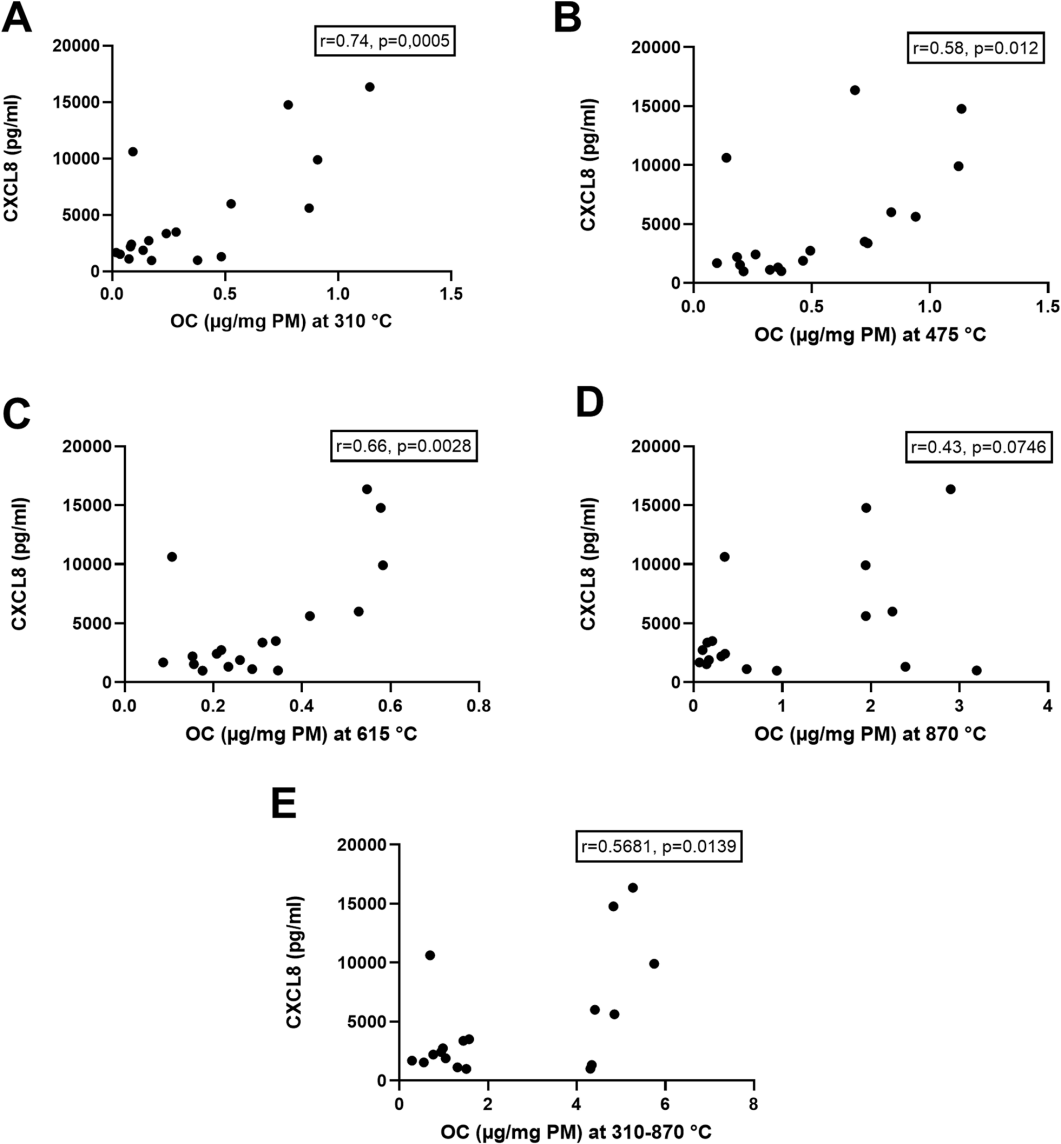
Correlation analysis between CXCL8 responses in HBEC3-KT cells and the content of organic carbon (OC) from coarse, fine and ultrafine PM sampled in road tunnels. The CXCL8 release at an exposure concentration of 100 µg/mL (10.4 µg/cm^2^) (n = 5) was compared to the levels of OC in the PM samples. The correlation was determined using Pearson’s correlation coefficient, which is presented in each panel. **A** OC at 310 °C; **B** OC at 475 °C; **C** OC at 615 °C; **D** OC at 870 °C; **E** OC at temperatures from 310 to 870 °C

Linear correlation analyses were carried out to assess putative correlations between different characteristics of the PM samples and pro-inflammatory responses in the HBEC3-KT cells. Overall, similar patterns were observed for all the different cytokines after exposure to the PM samples, with significant correlations between CXCL8 and IL-6 (r = 0.948, *p* < 0.00001); between CXCL8 and IL-lα (r = 0.909, *p*< 0.00001); and between CXCL8 and IL-lβ (r = 0.891, *p* < 0.00001) (see also, Additional file 5: Fig. S5). Thus, only the CXCL8 responses were used for further analyses of the correlation between pro-inflammatory potential and the different PM characteristics. Correlation analysis showed that the OC levels in the coarse, fine and ultrafine fractions of the sampled PM were correlated to the CXCL8 responses in the HBEC3-KT cells. The correlation coefficient depended on the evaporation temperature of different OC-fractions, and the strongest correlation was observed for the content of OC evaporating at 310 °C (r = 0.74, *p* = 0.0005) and the lowest for OC evaporating at 870 °C (r = 0.43, *p* = 0.0746). The correlation to total OC from 310 to 870 °C was relatively low (r = 0.568, *p* = 0.0139) (Fig. 6). No significant correlations were detected between particle-induced CXCL8 responses in the HBEC3-KT cells and the particle hydrodynamic size distribution (as measured on a mass basis), endotoxin content or oxidative potential (measured by ESR) (Additional files 6: Table S6).

## Discussion

In the present study, we assessed the pro-inflammatory effects of traffic-derived coarse, fine, and ultrafine PM sampled from two road tunnels paved with different stone materials during winter season. Sampling of PM in tunnels allows more selective studies of traffic-derived PM by minimizing contamination of particles from non-traffic sources. Such studies are scarce, but some studies have shown physicochemical characterization and source apportionment of road tunnel PM with relatively high levels of wear particles versus exhaust particles with varying content of asphalt abrasion, tire and brake wear PM, depending on season and geographical location [48, 49]. The pro-inflammatory responses of road tunnel PM have been even less characterized, with exception of our previous study [45]. In the present study, fine PM samples were more potent than the coarse samples, as assessed in human bronchial epithelial lung cells (HBEC3-KT), whereas the ultrafine samples varied more in potency between tunnels and road surface conditions. When comparing PM samples from the two tunnels under equal conditions, fine PM from Marienborg tunnel was more potent than fine PM from the Hell tunnel, whereas the coarse PM from the two tunnels showed more similar potencies. The pro-inflammatory potentials of the road tunnel PM were not correlated to hydrodynamic size distributions, endotoxin content or acellular ROS generation. However, the pro-inflammatory responses were positively correlated to OC-content. The stone materials used in the pavement of the two tunnels induced lower pro-inflammatory responses than the tunnel PM samples, particularly compared to fine PM sampled during humid conditions.

### Sampling and characterization of coarse, fine and ultrafine road tunnel PM

The relative mass levels of sampled PM differed between the road tunnels and were particularly high in Hell tunnel during dry conditions. This is in accordance with the longer tunnel length, higher number of vehicles per hour, higher speed limit and larger percentages of heavy vehicles and vehicles with studded tires compared to the Marienborg tunnel. The lower levels of PM sampled in Marienborg tunnel compared with the Hell tunnel could also be due to the ventilation system, which is better adjusted to the number of vehicles passing. As previously reported for ambient PM during winter season in Nordic countries, the mass of coarse PM tended to dominate over fine and ultrafine PM [41]. Humid road surface conditions decreased the levels of PM sampled in the Hell tunnel, in accordance with previous studies showing that road surface moisture affects particle mass levels [50].

The measurements of hydrodynamic size distribution showed that the fine and the ultrafine PM samples contained a large proportion of nano-sized particles (around 100 nm) related to mass, while these particles constituted a small proportion of the mass in the coarse PM samples. However, nano-sized particles dominated all size fractions on a particle number basis. Although, the road surface conditions seemed to induce some changes in these hydrodynamic size distributions, both for humid versus dry conditions, and with and without road surface cleaning, the changes were not consistent. Furthermore, it should be noted that the hydrodynamic size distributions of extracted PM samples may have limited value for assessing changes in the aerodynamic size distribution of PM in air.

The relative OC levels of total PM mass were higher in the fine than coarse PM samples, constituting around 50–60% and 10–15% of the total mass of the respective size-fractions. This may reflect that coarse PM is usually dominated by road dust which consists primarily of mineral particles [51, 52], while fine and ultrafine PM consist primarily of particles from combustion sources [26]. The OC content in the PM samples seemed to be relatively high in comparison to other studies reporting ratios of OC/PM in air from European cities for PM_2.5_ and PM_10_ [53, 54]. The large contribution of OC in our study could possibly be explained by lower temperatures in the tunnels/Nordic countries during winter due to increased binding of volatile or semi-volatile species to particles (secondary OC) [55–57]. This might also explain the higher content of OC in the PM sampled from the tunnel with lowest temperature, as the temperatures differed considerably inside the Marienborg and the Hell tunnel, with average temperatures of 7.3 °C and 14.9 °C, respectively. An unexpected finding in the present study was the very low levels of EC in comparison to OC in our PM samples, with EC levels below 1% of total carbon. In comparison, previous studies show much higher EC levels in traffic-related PM (10–30% of total carbon), depending on geographical location, distance to highways, influence from other PM sources, such as wood- and coal combustion, and seasonal differences in temperature and wind conditions [55, 56, 58–61]. The EC discrepancy could partly be due to different methods used, as peak inert mode temperatures used in present study (NIOSH, 870 °C) is much higher than in the Interagency Monitoring of Protected Visual Environment (IMPROVE, 550 °C) method. Thus, NIOSH may be subject to premature EC evolution (i.e., underestimation of EC), while IMPROVE may overestimate EC following incomplete OC evolution in the inert atmosphere [62]. Furthermore, we cannot exclude an insufficient extraction of EC from the particle filters. Nevertheless, it may be that the low EC levels reflect the low contamination with particles from other sources than traffic when sampling PM in a road tunnel. It may also be indicative of a high concentration of more volatile secondary OC [57]. The release of PM from modern cars has also been substantially reduced the recent years due to improved filter and catalysator technology. Thus, a particular reduction in EC, will shift the EC/OC ratio [63]. Furthermore, PM released from bitumen in asphalt which probably have a very low EC/OC ratio, may add to this finding.

### Pro-inflammatory responses of road tunnel PM compared to the respective stone materials in the pavement

In our early studies, feldspar content seemed to be inversely correlated to the pro-inflammatory potential of stone and mineral particles [45–47]. This has also found support in a recent clinical study where human volunteers exposed to quartz diorite particles showed stronger responses in terms of systemic markers of inflammation and blood coagulability than those exposed to rhomb porphyry particles with higher content of feldspar minerals [64, 65]. Furthermore, the clinical study also showed a stronger response for exhaled nitric oxide in volunteers exposed to quartz diorite than rhomb porphyry [66]. Conversely, recent in vitro studies show that stone particles samples composed primarily of feldspar minerals vary in potency, suggesting that other particle components or properties are more important for the pro-inflammatory potential of the particles [44]. Based on our early in vitro and in vivo studies, the Marienborg tunnel was paved with asphalt containing rhomb porphyry, a stone material that consists primarily of feldspar minerals, while the Hell tunnel was paved with quartz diorite, which contains larger amounts of quartz in addition to feldspar and other minerals [44]. While few significant differences were detected between the responses to coarse and fine PM samples from the two tunnels, significantly higher CXCL8 release was detected after exposure to fine PM from Marienborg tunnel compared to the Hell tunnel sampled during dry conditions. Thus, these results support that feldspar content is not a major determinant for the pro-inflammatory potential of traffic-related PM.

Overall, our results show that the PM sampled in the road tunnels mostly induced markedly higher pro-inflammatory responses than the responses induced by the respective stone materials used in the asphalt, suggesting that the stone materials alone contribute relatively little to PM reactivity. In support of this, Gerlofs-Nijland et al. showed that the pro-inflammatory potency of PM from tires and road wear by inhalation in mice was lower than for several other PM sources, including PM from brake pads and diesel exhaust [67]. In contrast, a previous study from the Hell tunnel showed that the mylonite material used in the pavement, which is the same as quartz diorite used in the present study, induced higher pro-inflammatory responses in lung cultures than road dust collected by an air filter in the ceiling of the tunnel [45]. The reason for this discrepancy is unclear but may be due to the sampling methodology or extraction procedure. It should be noted that a recent study from our research group showed that the rhomb porphyry and quartz diorite samples used in the present study induce similar pro-inflammatory responses that are lower than for several other stone particle samples [44]. Thus, it cannot be excluded that using a more potent stone material in the asphalt would lead to more potent road wear particles. In line with this, studies with particles generated in a road wear simulator suggest that wear particles containing asphalt particles with different stone materials differ in potency [24]. It should be emphasized that the potency of traffic-derived PM presumably is not due to a single source, but to a combined effect between PM from different sources, such as combustion PM and wear particles from the stone material, bitumen, tires and brakes. In a recent in vitro study we have shown that mineral particles increase the pro-inflammatory effects of diesel exhaust particles when administered in combination, suggesting that the impact of road wear particles may be larger than would be assumed from the potency of the stone particles alone [68].

### Potential effects of other sources on PM reactivity

In addition to particles from the stone material, other forms of wear particles, as well as particles from salting and sanding of the roads, may constitute a large part of the PM mass and potentially affect the PM reactivity. The relative content of salt in traffic-derived PM_10_ has been estimated to vary from 6–16% [69], and 2–8% [51] during the winter season in Nordic countries. Also the content of particles from sanding in traffic-derived PM_10_ is reported to be large, constituting 15–45% of PM mass in Nordic countries during winter, although lower than the levels of particles from the road pavement [51]. However, salting and sanding were not used inside the road tunnels in Trondheim, suggesting that these sources are minor contributors to the PM reactivity of our road tunnel PM samples. Conversely, particles from tires and brakes, which contains OC and different metals, may constitute a varying, but smaller part of PM2.5 and PM10 [70]. Although some experimental studies indicate a higher inherent reactivity of particles from these wear sources, further studies are needed to assess their respective contribution to the health impact of traffic-derived PM [67, 70]. The role of these particle sources has not been addressed in the present study of road tunnel PM.

### The importance of different PM characteristics for the differential pro-inflammatory responses of coarse, fine and ultrafine road tunnel PM

Overall, fine PM was more potent than coarse PM. Ultrafine PM was also less potent than the fine PM, with exception of the ultrafine PM from the Marienborg tunnel sampled during humid road surface conditions, that showed high responses. No correlations were observed between particle-induced CXCL8 release and the particle hydrodynamic size distributions on a mass basis, suggesting that the particle size was not a major determinant for the pro-inflammatory responses. However, it should be noted that firm conclusions on the impact of particle size on human health cannot be drawn as the hydrodynamic size distribution may not fully correspond to the aerodynamic size distribution of the particles in air. Likewise, no correlation was observed between PM-induced responses and endotoxin content, and exposure to LPS concentrations far higher than the ones detected in the PM samples also failed to elicit cytokine release from the HBEC3-KT cells, suggesting that these cells have low responses to endotoxin. This is in accordance with previous studies showing that very high concentrations of endotoxin are needed to induce pro-inflammatory responses in lung epithelial cells [39, 40]. In contrast to previous studies [10, 11], no correlation was observed between cytokine release and the ability to generate acellular ROS in the present study. While this suggests that ROS generated directly at the particle surface is not an important determinant for particle-induced cytokine release, this does not exclude the possibility that PM-induced cellular ROS via NADPH oxidase, mitochondrial damage or CYP metabolism may be involved in pro-inflammatory responses and cytotoxicity [71].

Notably, a significant correlation was observed between pro-inflammatory responses and OC content of the particle samples, suggesting OC to be an important contributor to the reactivity of the tunnel PM. The correlation was most pronounced for the most volatile OC fraction evaporated at 310 °C. The pro-inflammatory responses to the PM sampled in the Marienborg tunnel were higher than in the Hell road tunnel, and this is presumably related to lower temperatures, which cause less evaporation of OC and higher amounts of OC attached to PM. Interestingly, the lower pro-inflammatory activity of ultrafine PM from the Marienborg tunnel sampled after road surface cleaning occurred in parallel to a reduction of OC. Conceivably, the role of OC is attributed to PAHs that are important constituents of OC in ambient PM. The PAHs seem to have an important role in PM-induced inflammation and adverse effects in the airways and cardiovascular system [26, 72]. However, further studies are needed to establish the causal relationship between OC/PAHs and the potential of the traffic-related PM to induce pro-inflammatory responses.

### Conclusions, methodological considerations and further perspectives

The present study shows that traffic-related PM sampled in two road tunnels induced marked pro-inflammatory responses in bronchial epithelial lung cells in vitro, with fine PM as the most potent. The pro-inflammatory responses induced by particles from the stone materials used in the pavement were substantially lower than that of the road tunnel PM on an equal mass basis, suggesting that the stone material alone is not a major determinant for the high reactivity of traffic-related PM. However, we cannot discount the possibility that combinative effect between combustion PM and mineral, or other PM constituents, contribute to the reactivity of PM. Importantly, our characterization of the PM samples is limited and should be extended with further analyses of particle morphology, as well as analyses of metals and specific chemical groups to elucidate their potential role. Further studies are also warranted regarding the potential role of combustion PM for the pro-inflammatory outcome of road tunnel PM, alone and in combination with stone materials. Notably, the ratio between OC and total PM mass seemed important for the PM-induced pro-inflammatory responses. This ratio can be influenced by various factors including temperatures, OC sources and the amount of stone particles from abrasion of road pavement. Whether the OC originates from fuel combustion, bitumen from asphalt, or from tires, may have important implications for which preventive measures are most relevant from a health perspective. It will be crucial to further substantiate any possible causal relationship between the OC and pro-inflammatory outcome for road tunnel PM both in in vitro and in vivo. Finally, it should be noted that that particle dosimetry and environmental concentrations have to be taken into account to examine whether the in vitro findings of road tunnel PM are relevant for assessment of the actual impact on human health.

## Materials and methods

### Materials

The cell culture medium LHC-9, DMEM:F12 and Trypsin–EDTA were bought from Gibco, Thermo Fisher Scientific, Waltham, MA, USA. The cell culture flasks were obtained from Nunc A/S, Roskilde, Denmark, while the cell culture plates were from Corning, NY 14831 USA. PureCol collagen was from Advanced BioMatrix, Inc, CA, USA. The Cytotoxicity Detection Kit using lactate dehydrogenase (LDH) activity was bought from Merck KGaA, Darmstadt, Germany. AlamarBlue™ Cell Viability Reagent and the sandwich enzyme-linked immunosorbent assay (ELISA) cytosets for CXCL8 and IL-6 were purchased from Invitrogen, Thermo Fisher Scientific, Waltham, MA, USA, while IL-1α and IL-1β DuoSet were from R&D Systems, Inc, Minneapolis, MN, USA. The Endotoxin Quant kit were from Thermo Fisher Scientific, Waltham, MA USA. For measurements of acellular ROS 5,5-dimethyl-1-pyrroline-N-oxide (DMPO), Dulbecco’s PBS; hydrogen peroxide (H_2_O_2_), dithiothreitol (DTT) and ultrapure H_2_O were purchased from Merck KGaA, Darmstadt, Germany. Whatman TE 38 filter was from (Whatman, Maidstone, Kent, UK). Other chemicals were purchased from commercial sources at the highest purity available.

### Description of road tunnels

In the present study, coarse PM samples (between 10 and 2.5 µm), fine PM samples (between 2.5 and 0.18 µm) and ultrafine PM samples (below 0.18 µm) were collected inside two different road tunnels in Trondheim, Norway during late winter season (March 2019). The two tunnels, Marienborg tunnel and Hell tunnel, were paved with SMA16 asphalt containing the stone materials rhomb porphyry and quartz diorite, respectively, also referred to as rock aggregates. The rhomb porphyry has a ball mill value A_N_ = 7.5 and Los Angeles value LA = 16.4 which means it can be used as aggregates in a surface course on roads with annual average daily traffic (AADT) 5000–15,000 according to existing requirements. The quartz diorite has a ball mill value A_N_ = 5.2 and Los Angeles value LA = 10.3 which means it can be used as a surface course on roads with AADT > 15,000. Both tunnels have one tube with one lane in each direction and a ventilation system consisting of large mechanical fans mounted on the ceiling. The principle is to ventilate the tunnels longitudinally in the dominant wind direction, but this can be hindered by the opposing traffic that could affect the flow of air and keep the pollution in the middle of the tunnel. The Marienborg tunnel was opened in 2010 and is located in Trondheim on RV706 and has a speed limit of 60 km/h. It is 1840 m long with a 3-way roundabout at the northern end and is aligned in the north–south direction. The PM sampling was performed at an emergency bay beside the traffic lane inside the road tunnel, approximately 780 m from the southern entrance. The AADT is approximately 7000 vehicles per 24 h, with 10% heavy vehicles. The proportion of vehicles using studded tires was around 25% in the period PM was sampled. The average temperature in the tunnel during the whole sampling period was 7.3 °C, and the relative humidity was 83.2%. The Hell tunnel was opened in 1995 and is located outside Trondheim on EV6. It has a speed limit of 80 km/h and is 3924 m long with a grade of 2.3%. The PM sampling was performed in an emergency parking area inside the tunnel approximately 2140 m from the southern entrance. The AADT is approximately 16,000 vehicles each 24 h, with 17% of heavy vehicles. The percentage of vehicles with studded tires was approximately 40% in the sampling period. The average temperature in the tunnel was 14.9 °C, and the relative humidity 60.5%.

### Sampling and preparation of road tunnel PM

Coarse, fine, and ultrafine particles were sampled with a vacuum pump and a high-volume cascade impactor sampler. Particles were sampled in the tunnels during dry and humid road surface conditions, and before and after cleaning of the road surface and side areas in the tunnels. Sampling was performed 10 days after a comprehensive washing of the tunnel, without any other cleaning in between before the PM sampling, and was performed at a flow rate of 900 L/h in 10–12 h periods. Visual status of road surface was registered (humid and dry conditions). To assess the effect of cleaning an ordinary routine road surface cleaning was performed immediately after sampling of PM from the non-cleaned surface. Such cleanings are done in the same way in both tunnels by the same contractor, using a RotorClean system with high pressure water and suction. A rod with high pressure nozzles is used to move the road dust from the 0.5 m lower wall, side areas and curbsides for the machine to be able to collect the road dust with the high vacuum suction. For PM sampling we used a high-volume cascade impactor sampler developed by Demokritou et al. [73], with a multistage round slit nozzle, and polyurethane foam (PUF) used as impaction substrate for the coarse and fine PM, and a Whatman TE 38 filter for the ultrafine PM. Pre-weighted clean PUFs and filters were stored in closed containers in a dark room before use. After PM collection, both PUFs and filters were sealed in the original container and stored in refrigerator until further analysis. PUFs and filters were placed in a conditioned room with steady temperature and humidity for 24 h and weighed on an analytical balance.

The collected PM samples were extracted from the PUFs and filters in 50 mL plastic tubes by adding about 25 mL methanol, followed by thorough mixing on a vortexer and 2–5 min sonication in a water bath (Elmasonic S) to dislodge the particles from the filters. The particle suspensions were then transferred to a glass flask and the extraction was repeated until most of the particles was removed from the PUFs/filters. Most of the methanol was removed by a rotary evaporator before the PM solutions were transferred to pre-weighted glass flasks and kept at 25 °C to remove the rest of the methanol. The weights of the extracted particles were determined gravimetrically [14]. The mean efficiency was 50%. The particle samples were suspended in pyrogen-free sterile water to a concentration of 10 mg/mL and sonicated for 5 min before freezing at − 20 °C. Before use the PM were mixed well and re-sonicated for 1 min.

### Characterization of road tunnel PM

The PM samples were characterized by hydrodynamic size distributions, endotoxin content, the ability to generate acellular ROS, and the of content EC and OC.

#### Hydrodynamic size distribution

The hydrodynamic sizes of tunnel PM samples were determined by using a CPS Disc Centrifuge model DC 24000 (Inventech Benelux, Oosterhout, the Netherlands https://www.inventech.nl).

#### Measurements of endotoxins

The endotoxin content of all the PM samples (100 µg/mL) was measured by the Pierce Chromogen Endotoxin Quant kit (LAL-method), using an endotoxin-standard from 0.01 to 0.1 endotoxin units (EU)/mL. The kit’s procedure was followed with minor alterations. As the turbidity of the particles would interfere with the assay, the plate was centrifuged at 3220×*g* to pellet the particles immediately after the stop solution was added to the reaction mixture. The supernatants (100 µL) were transferred to a clean 96-well plate and the optical densities (OD) at 405 nm were measured. The average absorbance of the blank replicates was subtracted from the average absorbance of all individual standards and sample replicates to calculate mean absorbance. A standard curve was prepared without the blank, R^2^ was ≥ 0.98. The formulated standard curve (linear regression) was used to determine the endotoxin concentration of each sample.

#### Acellular reactive oxygen species/oxidative potential

Acellular ROS was measured by OP^ESR^ on a MiniScope MS 400 (MT MagnetTech Gmbh, Berlin, Germany) and by OP^DTT^ on a regular spectrophotometer. OP^ESR^ is a spectrophotometric method using DMPO as a spin trap in the presence of H_2_O_2_, measuring hydroxyl radical generation (OH∙) by the PM samples, and is mainly sensitive to metal-mediated ROS formation by Fenton-type-reactions [74]. The radical generation was measured in triplicates on the samples (25 µg) after 15 min, with ultrapure water used as control and Diesel Oil Fly Ash (DOFA) as a positive control. The DTT assay measures the presence of ROS via formation of DTT disulfide due to transfer of electrons from DTT to oxygen, based on the average of two duplicate readings, resulting in a value expressed as nmol DTT consumed per min per μg sample [10]. In the DTT analyses we used 5 µg of each sample, the signal was measured after 0, 10, 20 and 30 min.

#### OC and EC analyses

The OC and EC content of tunnel PM samples were analyzed by a method adopted from NIOSH temperature protocol allowing to distinguish different temperature fractions (i.e., volatility and molecular weight as previously described [75–77] using thermal optical analyzer (Sunset Laboratory, Inc.). For analysis, 5 µL aliquots of PM solutions (10 mg/ml) were placed onto pre-baked quartz filters (600 °C, overnight) and dried on a hot plate set to 50 °C for 7 min. Laser transmittance at 658 nm was used to distinguish pyrolyzed OC from EC. The temperature profile began with a 10 s ambient temperature step followed with 5 steps; 300 °C for 75 s, 500 °C for 75 s, 600 °C for 75 s, 700 °C for 75 s, and 870 °C for 120 s, in a helium atmosphere. This was followed by a cooling step to 525 °C for 45 s. Five additional temperature steps were followed in a 5% oxygen in helium atmosphere; 550 °C for 45 s, 625 °C for 45 s, 700 °C for 45 s, 775 °C for 45 s, and 890 °C for 120 s. Lastly, a calibration gas of 5% methane/helium was added as an internal standard for 110 s. The total analysis time was 885 s. To test for a potential occurrence of carbonate, which could interfere with OC measurements, samples were treated with HCl. No loss of carbon was observed indicating no significant amounts of carbonates.

### Preparation, size distribution and mineral composition of stone materials

Two stone materials, rhomb porphyry and quartz diorite, were included in the present study, and correspond to the materials used in the pavement of the Marienborg and Hell road tunnels, respectively. The preparation of these stone particle samples with sizes < 10 µm has been described in a recent study [44]. Before use, the particles were suspended in pyrogen-free sterile water at a concentration of 10 mg/mL and sonicated for one min in a water bath (Elmasonic S). The mineralogical and elemental composition of the samples has previously been described [44]. The rhomb porphyry sample contained the highest amount of feldspar minerals, in the form of K-feldspar and plagioclase, in addition to smaller amounts of hornblende, chlorite, calcite, quartz and muscovite. In comparison, the quartz diorite sample contain larger amounts of quartz, in addition to K-feldspar, plagioclase, epidote, chlorite and muscovite. The elemental composition and endotoxin content were quite similar between the samples [44].

### Cell culture and exposure conditions

HBEC3-KT cells (passage 4-35, ATCC CRL-4051) were maintained in LHC-9 medium in collagen-coated flasks (PureCol) in a humified atmosphere at 37 °C with 5% CO_2_. The cell culture medium was replaced every second day as described in a previous study [9]. For experiments, the cells were seeded on pre-coated 6-well plates at a density of 170.000 cells/cm^2^ three days before exposure. The medium was changed to serum-free DMEM:F12 one day before exposure. The cells were exposed to the PM samples for 20 h in DMEM:F12. This early timepoint is often chosen for assessing acute toxicity in cell culture studies [9, 78].We also performed studies at 40 h, with similar results for the road tunnel PM (data not shown). Cell culture media were collected and centrifuged at 300×*g* to remove cellular debris and followed by centrifugation at 10.000×*g* to remove floating particles. The samples were then frozen at − 80 °C awaiting analysis.

### Cytokine analysis

After thawing the frozen samples, the concentrations of CXCL8, IL-6, IL-1α and IL-1β were determined by ELISA according to the manufacturer’s guidelines. Absorbance was measured and quantified by a plate reader (TECAN Sunrise, Männedorf, Switzerland) equipped with a dedicated software (Magellan V 1.10), as previously described.

### Cell viability

Cell viability was measured using the AlamarBlue assay and release of LDH. In the AlamarBlue assay the metabolic activity was analyzed as described in the producer’s manual after 20 h of exposure using a CLARIOstar plate reader (BMG LABTECH, Ortenberg; Germany). The LDH concentration was measured in media after 20 h according to the manufacturer’s guideline (Roche, Germany).

### Statistical analyses

Statistical analyses were performed by using GraphPad Prism software (version 9.0 Inc., San Diego, CA). Statistically significant differences were determined using either one-way repeated measures analysis of variance (ANOVA) with Tukey’s multiple comparisons test or two-way repeated measures ANOVA with Dunnett’s or Tukey’s multiple comparison tests, depending on the experimental design. A repeated measures design, in which all values belonging to the same experiment were defined as a subject, was used to reduce the impact of between-experiment variation not due to the experimental treatment. Experiments were performed in 3–7 independent biological replicates. Based on the evaluation of QQ-plots several data were log-transformed to ensure normality. Geisser–Greenhouse correction was used to account for non-sphericity in the data. The correlations between particle size, endotoxin content, acellular ROS and organic content and the ability to induce pro-inflammatory responses in HBEC3-KT were determined using Pearson’s correlation coefficient. To assess the correlation between particle size and pro-inflammatory responses, area under the curve (AUC) values were calculated from either the full-size distribution curves, or from specific segments of the curves, using the trapezoid method.

## Supplementary Information

**Additional file 1: Fig. S1**. Geographical location of the Marienborg and Hell road tunnels in the Trondheim area in Norway.

**Additional file 2: Fig. S2**. Hydrodynamic size distribution based on particle number of coarse, fine and ultrafine PM sampled in the Marienborg and Hell tunnels. A) Marienborg PM dry road surface conditions; B) Marienborg PM humid road surface conditions; C) Hell PM dry road surface conditions; D) Hell PM humid road surface conditions. The hydrodynamic size distributions were determined by the Disc centrifugation method as described in Materials and Methods.

**Additional file 3: Fig. S3**. Correlation plots between potential for generation of acellular ROS as measured by ESR (OP^ESR^ ) and by DTT (OP^DTT^) methods. The correlation was determined using Pearson’s correlation coefficient.

**Additional file 4: Fig. S4**. Cytokine responses in HBEC3-KT cells after exposure to PM samples from two road tunnels before and after road surface cleaning. The cells were exposed to PM samples from the Marienborg and Hell tunnel during humid and dry road surface conditions, respectively, and compared to PM sampled after road surface cleaning. The cells were exposed to 100 µg/mL (10.4 µg/cm2) for 20 h. The cytokine release was analysed by ELISA. A) CXCL8; B) IL-6; C) IL-1α; D) IL-1β. The data represent the mean +/- SEM of 3-5 experiments. # Significantly different from PM samples before cleaning p < 0.05.

**Additional file 5: Fig. S5**. Correlation matrix between CXCL8, IL-6, IL-1α and IL-1β release. The cytokine release was determined after an exposure to 100 µg/mL (10.4 µg/cm2) of all the particles (n = 5). The correlation was determined using Pearson’s correlation coefficients (r is listed in the matrix).

**Additional file 6: Table S6**. Correlation plots between particle characteristics and CXCL8 responses. The CXCL8 release at an exposure concentration of 100 µg/mL (10.4 µg/cm^2^) (n =5) was compared to hydrodynamic size distributions of the particles, endotoxin levels, generation of acellular ROS as measured by ESR (OP^ESR^ ) and by DTT (OP^DTT^) method, ; and the levels of OC as measured at 310 ºC; at 475 ºC; at 615 ºC; at 870 ºC, and at temperature from 310°C-870°C in the PM samples. The correlations were determined using Pearson’s correlation coefficients.

## Data Availability

The datasets used in the presents study are available from the corresponding author upon reasonable request.

## Abbreviations

AUC: Area under curve
CXCL8: Chemokine CXC-motif ligand 8
DEP: Diesel exhaust particles
DTT: Dithiothreitol
EC: Elemental carbon
ESR: Electro spin resonance
HBEC3-KT: Human bronchial epithelial cells
IL: Interleukin
LDH: Lactate dehydrogenase
LPS: Lipopolysaccharide
OC: Organic carbon
OP: Oxidative potential
PAH: Polycyclic aromatic hydrocarbons
PM: Particulate matter
ROS: Reactive oxygen species
SEM: Standard error of mean
TNFα: Tumor necrosis factor alpha
