# Supplementary figures and images for: Road tunnel-derived coarse, fine and ultrafine particulate matter: physical and chemical characterization and pro-inflammatory responses in human bronchial epithelial cells
Source: Part Fibre Toxicol. 2022 Jul 4;19:45. doi: 10.1186/s12989-022-00488-5 (PMC9251916; doi:10.1186/s12989-022-00488-5)

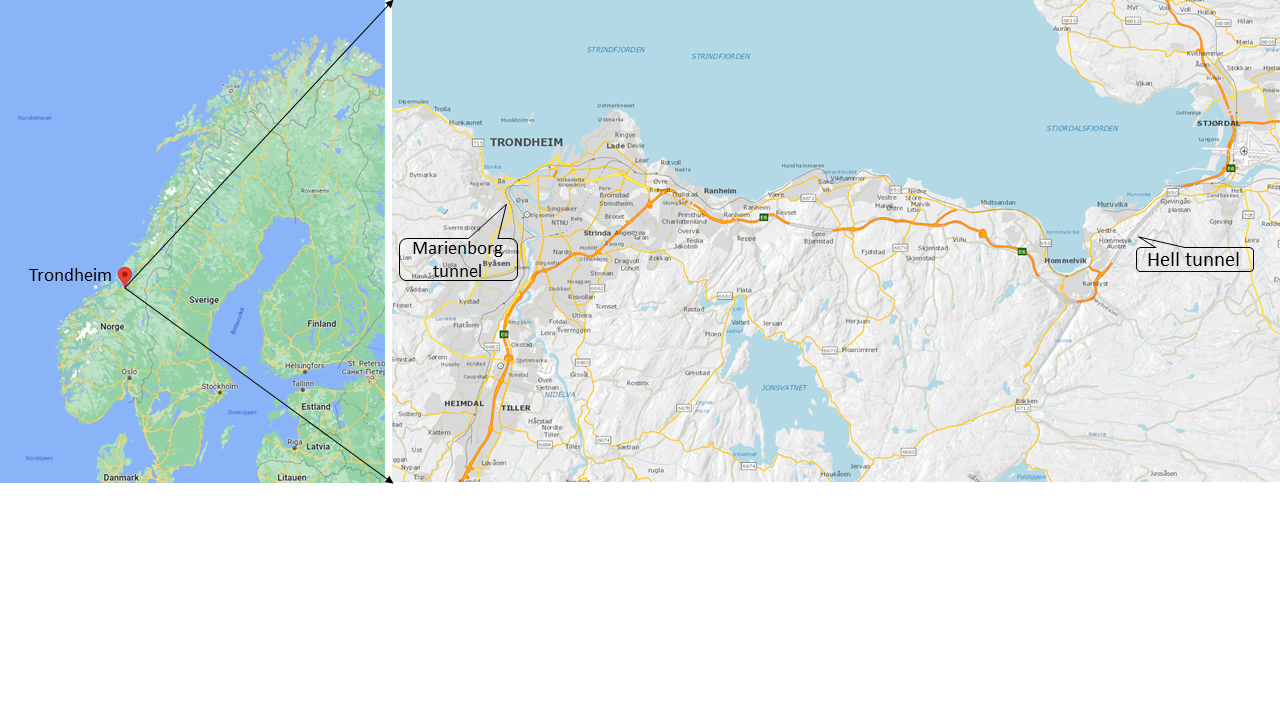

Supplement: Supplementary file 1 — Additional file 1: Fig. S1. Geographical location of the Marienborg and Hell road tunnels in the Trondheim area in Norway. [file 12989_2022_488_MOESM1_ESM.tif]

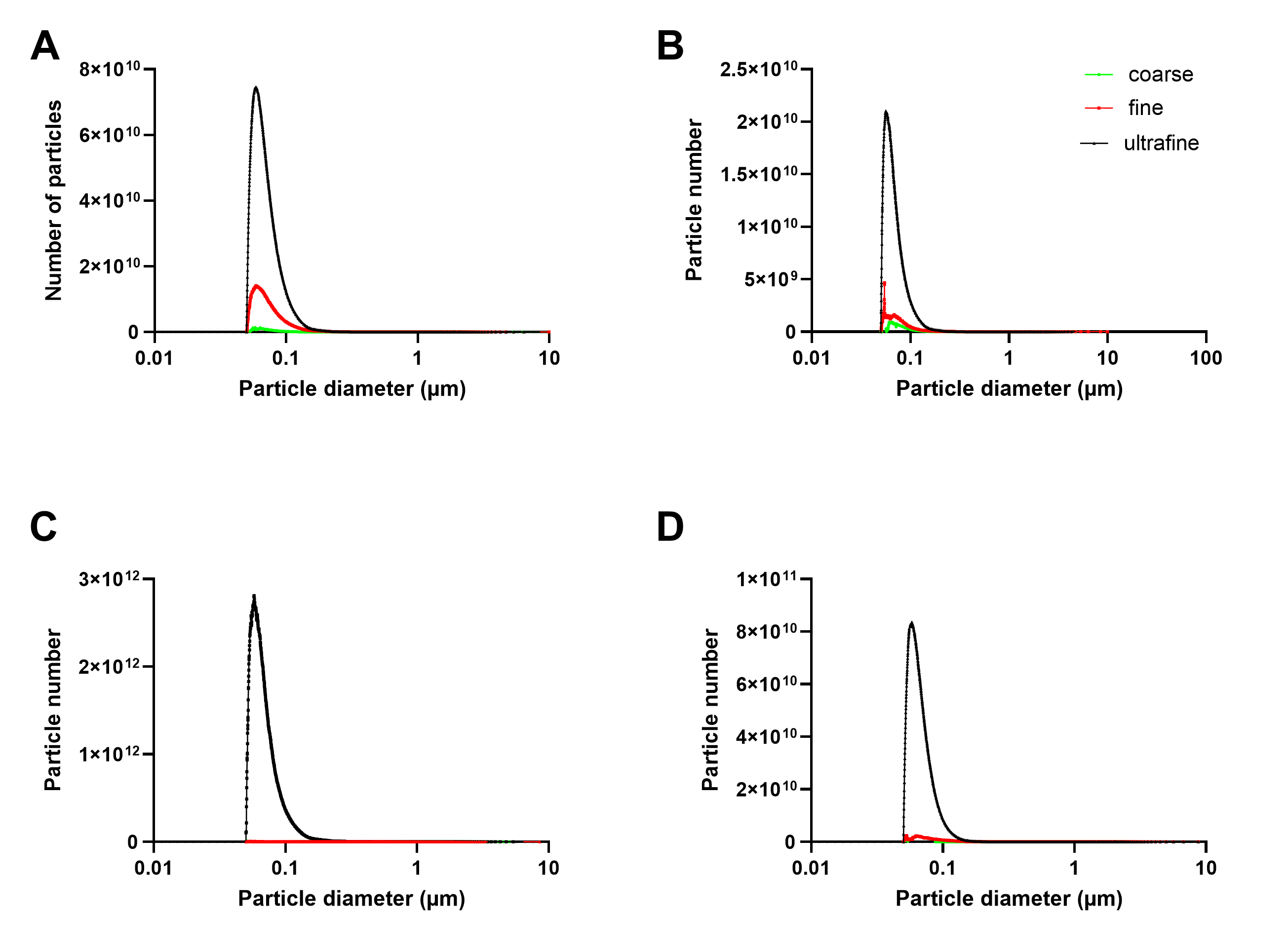

Supplement: Supplementary file 2 — Additional file 2: Fig. S2. Hydrodynamic size distribution based on particle number of coarse, fine and ultrafine PM sampled in the Marienborg and Hell tunnels. A) Marienborg PM dry road surface conditions; B) Marienborg PM humid road surface conditions; C) Hell PM dry road surface conditions; D) Hell PM humid road surface conditions. The hydrodynamic size distributions were determined by the Disc centrifugation method as described in Materials and Methods. [file 12989_2022_488_MOESM2_ESM.tif]

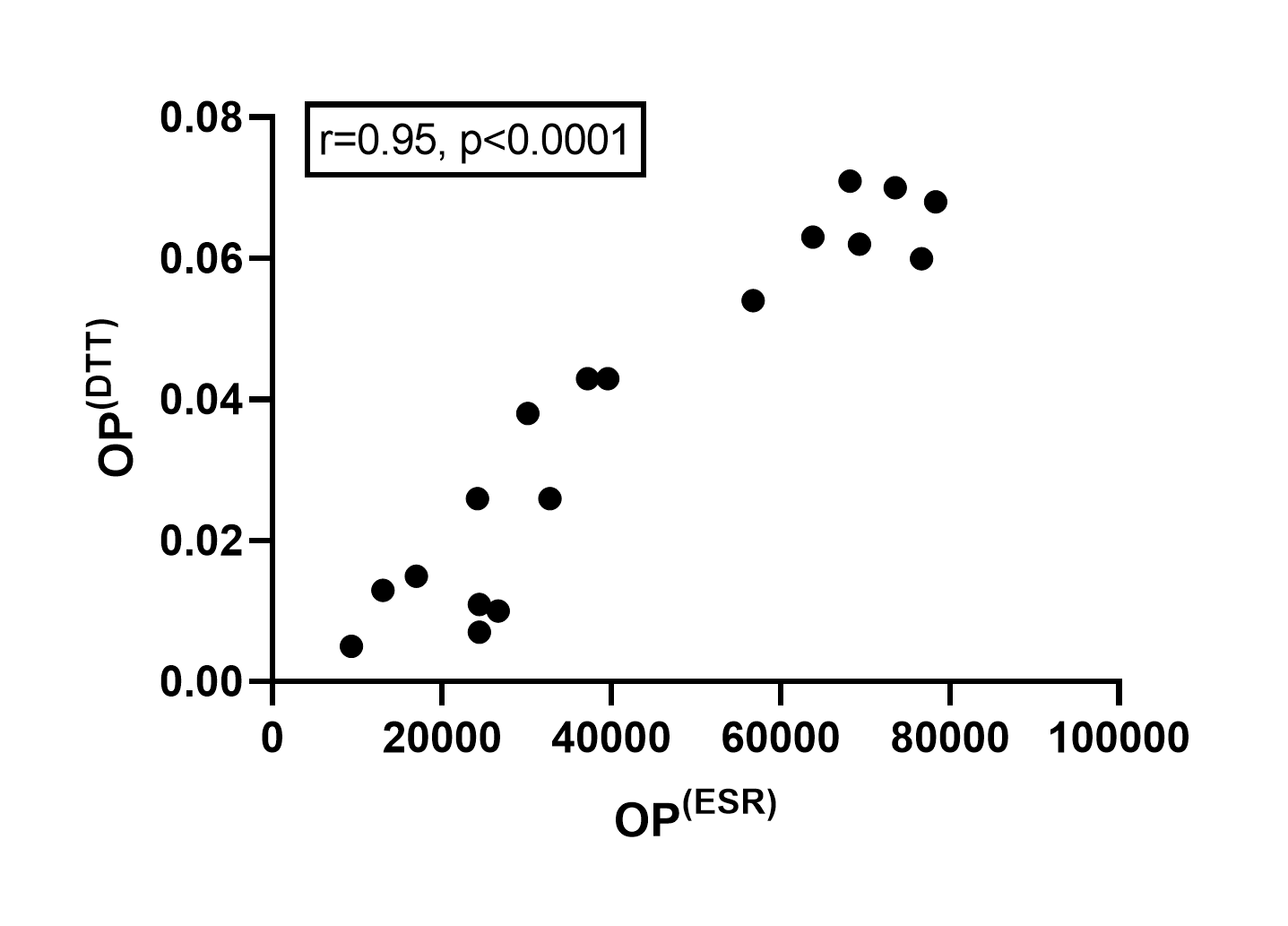

Supplement: Supplementary file 3 — Additional file 3: Fig. S3. Correlation plots between potential for generation of acellular ROS as measured by ESR (OPESR ) and by DTT (OPDTT) methods. The correlation was determined using Pearson’s correlation coefficient. [file 12989_2022_488_MOESM3_ESM.tif]

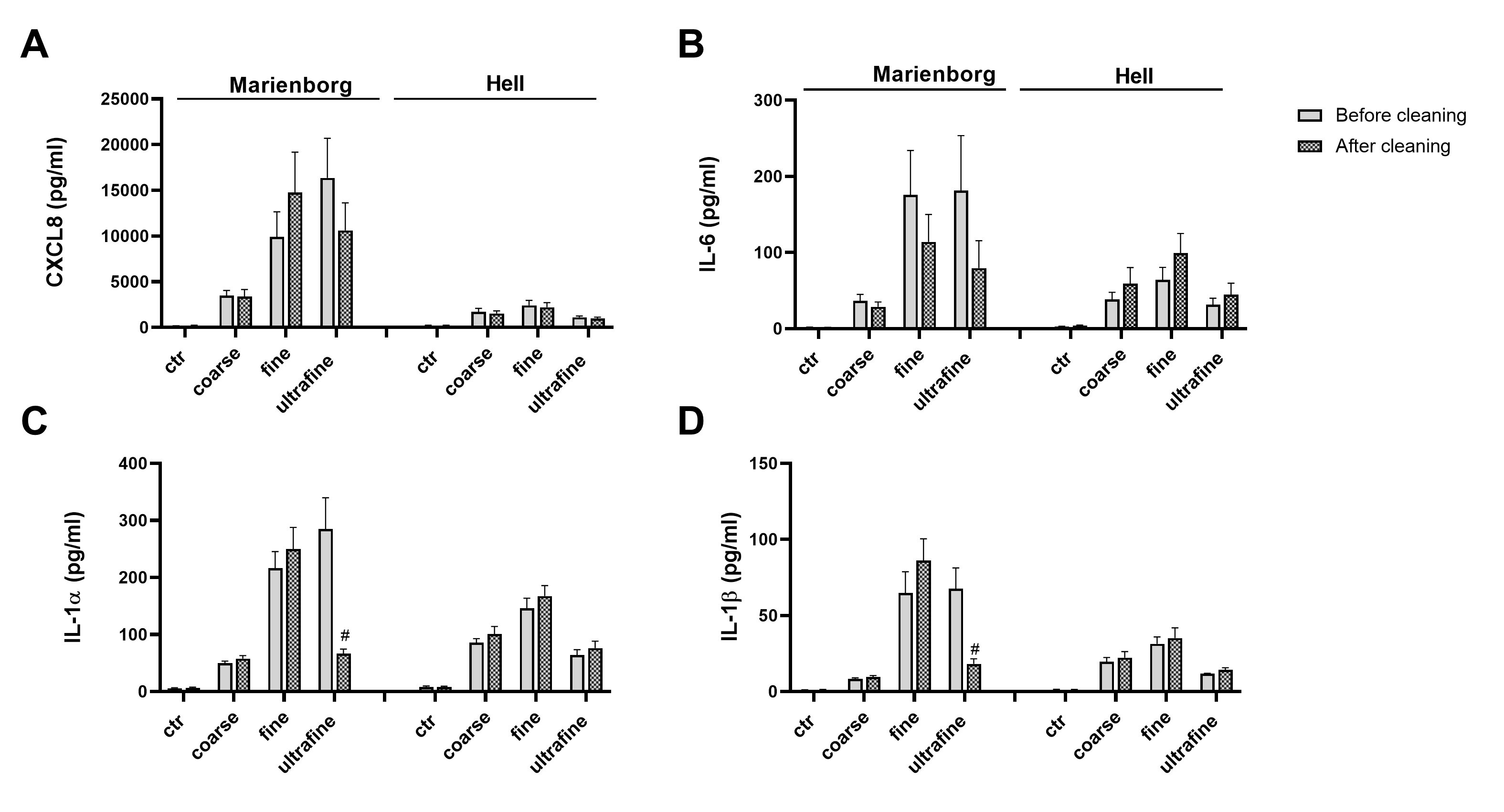

Supplement: Supplementary file 4 — Additional file 4: Fig. S4. Cytokine responses in HBEC3-KT cells after exposure to PM samples from two road tunnels before and after road surface cleaning. The cells were exposed to PM samples from the Marienborg and Hell tunnel during humid and dry road surface conditions, respectively, and compared to PM sampled after road surface cleaning. The cells were exposed to 100 µg/mL (10.4 µg/cm2) for 20 h. The cytokine release was analysed by ELISA. A) CXCL8; B) IL-6; C) IL-1α; D) IL-1β. The data represent the mean +/- SEM of 3-5 experiments. # Significantly different from PM samples before cleaning p < 0.05. [file 12989_2022_488_MOESM4_ESM.tif]

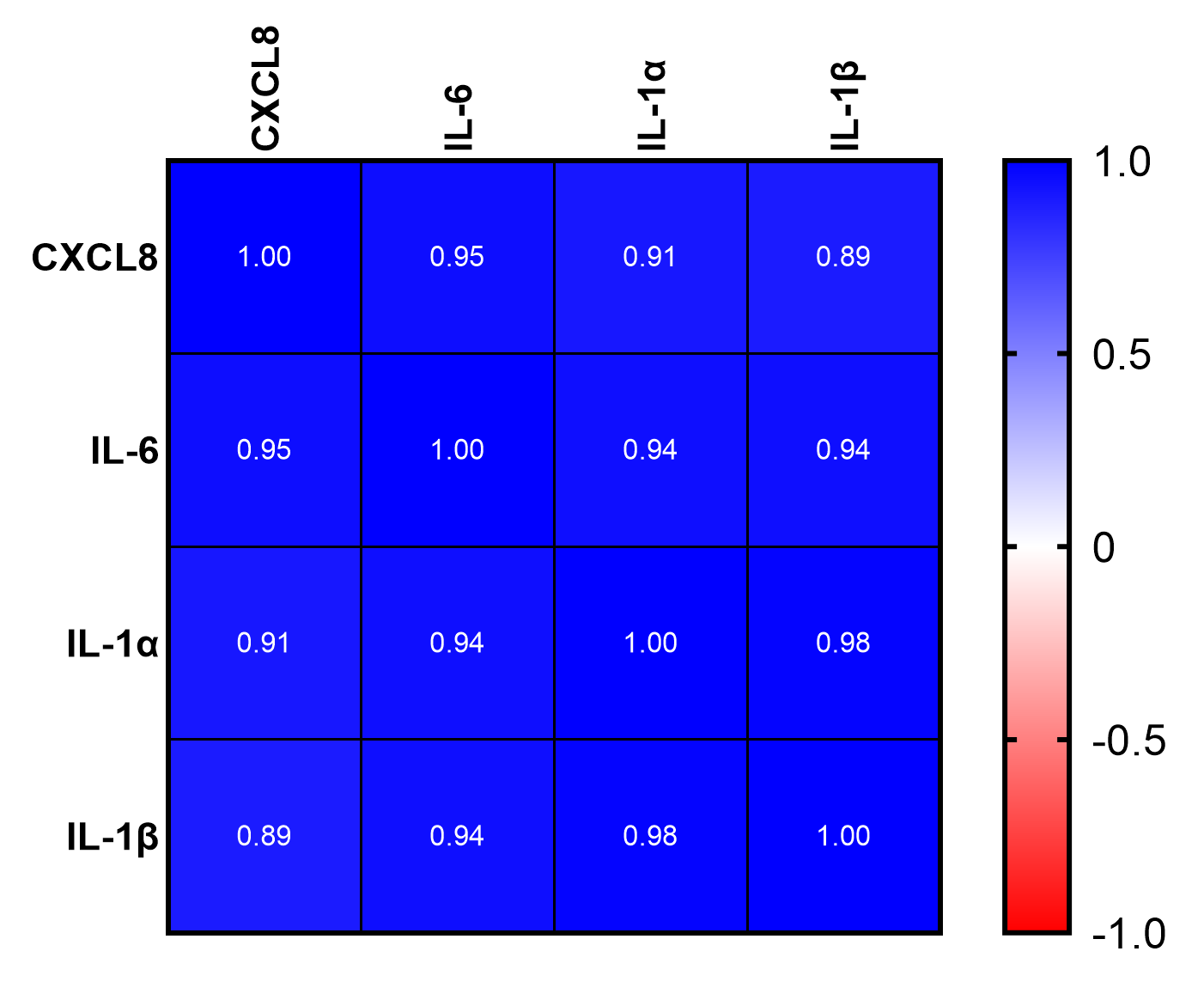

Supplement: Supplementary file 5 — Additional file 5: Fig. S5. Correlation matrix between CXCL8, IL-6, IL-1α and IL-1β release. The cytokine release was determined after an exposure to 100 µg/mL (10.4 µg/cm2) of all the particles (n = 5). The correlation was determined using Pearson’s correlation coefficients (r is listed in the matrix). [file 12989_2022_488_MOESM5_ESM.tif]
