# Supplementary material for: Road tunnel-derived coarse, fine and ultrafine particulate matter: physical and chemical characterization and pro-inflammatory responses in human bronchial epithelial cells
Source: Part Fibre Toxicol. 2022 Jul 4;19:45. doi: 10.1186/s12989-022-00488-5 (PMC9251916; doi:10.1186/s12989-022-00488-5)
Supplement: Supplementary file 6 — Additional file 6: Table S6. Correlation plots between particle characteristics and CXCL8 responses. The CXCL8 release at an exposure concentration of 100 µg/mL (10.4 µg/cm2) (n =5) was compared to hydrodynamic size distributions of the particles, endotoxin levels, generation of acellular ROS as measured by ESR (OPESR ) and by DTT (OPDTT) method, ; and the levels of OC as measured at 310 ºC; at 475 ºC; at 615 ºC; at 870 ºC, and at temperature from 310°C-870°C in the PM samples. The correlations were determined using Pearson’s correlation coefficients. [file 12989_2022_488_MOESM6_ESM.docx]

| **Correlation analysis between CXCL8-release and particle characterization** | | |
| --- | --- | --- |
| **Particle characterization** | **Pearson r** | **p value** |
| Hydrodynamic size diameter; 0-10 µm (AUC) | -0.11 | 0.655 |
| Hydrodynamic size diameter; <0.18 µm (AUC) | -0.11 | 0.668 |
| Hydrodynamic size diameter; 0.18-2.5 µm (AUC) | -0.06 | 0.811 |
| Hydrodynamic size diameter; 2.5-10 µm (AUC) | 0.25 | 0.309 |
| Endotoxin content | -0.31 | 0.330 |
| Reactive oxygen species | 0.29 | 0.230 |
| Organic Carbon from 310-870 °C | 0.57 | 0.014 |
| Organic Carbon at 310 °C | 0.74 | 0.001 |
| Organic Carbon at 475 °C | 0.58 | 0.012 |
| Organic Carbon at 615 °C | 0.66 | 0.003 |
| Organic Carbon at 870 °C | 0.43 | 0.075 |
